# Evaluation of the antibacterial activity of the natural product α-mangostin against *Clostridioides difficile*

**DOI:** 10.1371/journal.pone.0341857

**Published:** 2026-02-05

**Authors:** Brice J. Stolz, Ahmed Abouelkhair, Nader S. Abutaleb, Mohamed N. Seleem

**Affiliations:** 1 Department of Biomedical Sciences and Pathobiology, Virginia-Maryland College of Veterinary Medicine, Virginia Polytechnic Institute and State University, Blacksburg, Virginia, United States of America; 2 Center for One Health Research; Virginia Polytechnic Institute and State University, Blacksburg, Virginia, United States of America; Cornell University, UNITED STATES OF AMERICA

## Abstract

*Clostridioides difficile* is the leading cause of hospital-associated diarrhea and has remained a consistent threat for older patients and those with comorbidities or vulnerabilities. The high rates of treatment failure and recurrence, along with the decreased effectiveness of first-line treatments highlight the urgent need for the development of new anti-*C. difficile* agents. α-mangostin is a natural compound isolated from the edible mangosteen fruit pericarps that has known antimicrobial activity. α-mangostin is poorly absorbed from the gastrointestinal tract (GIT), which is ideal for treatment of CDI to accumulate at the site of infection at concentrations capable of clearing *C. difficile*. We found that α-mangostin was as potent as the standard-of-care vancomycin, inhibiting a diverse panel of *C. difficile* strains at a concentration range of 0.5–2 µg/mL. It exhibited rapid bactericidal activity, completely clearing *C. difficile in vitro* within 2 hours, surpassing vancomycin and fidaxomicin. Additionally, α-mangostin’s anti-*C. difficile* activity was not affected by the high *C. difficile* inoculum. To further understand its mechanism, we investigated α-mangostin’s membrane disruption activity by assessing the leakage of DNA and ATP post-exposure. α-mangostin resulted in a significant leakage of DNA and ATP indicating that its anti-*C. difficile* activity is mediated by the bacterial cell membrane disruption. Collectively, these findings demonstrate that α-mangostin possesses desirable characteristics for a promising anti-*C. difficile* which merits further investigation.

## Introduction

*Clostridioides difficile* infection (CDI) is a leading cause of hospital-associated infections and antibiotic-associated diarrhea. The incidence of CDI has increased due to the emergence of hypervirulent epidemic strains, such as the BI/NAP1/027 strain, which were responsible for several outbreaks worldwide, This strain is often associated with more severe disease and increased mortality rates [1–4]. Recently, it was reported by the CDC’s 2024 According to the recent Emerging Infections Program (EIP) report of the U. S. Centers for Disease Control and Prevention (CDC), the overall incidence rate of CDI was 116.1 cases per 100,000 individuals, with a higher incidence of community-associated cases (62.1 cases per 100,000 persons) compared with healthcare-associated cases (54.0 cases per 100,000 persons) [5].The majority of cases are within inpatient care and 80% of deaths occur in those who are 65 years and older [6,7]. CDI healthcare costs due to prolonged hospitalization and repeated antibiotic usage are estimated to be $5–6 billion per year in the United States [8]. The current mean treatment cost for CDI hospitalization is approximately $21,448 per case and varies widely based upon other illnesses such as renal impairment where costs may balloon to over $100,000 [8]. Therefore, The CDC classified CDI as an urgent public health threat due to its severe impact and high potential for widespread transmission and thus necessitating immediate action.

Currently, only two antibiotics are approved for the treatment of CDI: vancomycin and fidaxomicin [9,10]. Metronidazole was initially recommended for mild/moderate CDI. Yet, the new guidelines of the Infectious Diseases Society of America and Society for Healthcare Epidemiology of America (IDSA/SHEA) and the European Society of Clinical Microbiology and Infectious Diseases (ESCMID recommend metronidazole only if fidaxomicin and vancomycin are unavailable [11,12]. Other non-antibiotic therapeutics include monoclonal antibodies, like bezlotoxumab, targeting *C. difficile* toxins, and fecal microbiota transplantation (FMT) which targets recurrent infections [13]. However, the clinical outcomes of these therapeutics are not satisfactory. Vancomycin and fidaxomicin are compromised by the high rates of treatment failure and subsequent recurrence reaching as high as 30% and 20%, respectively [14,15]. Though fidaxomicin is a more effective therapeutic for CDI with lower recurrence rate, its use is limited by the high cost (about $38,000 for treatment depending on the severity of the infection) and a few reports indicated that it does not provide a significant benefit over vancomycin in patients with CDI caused by BI/NAP1/027 [16–20]. Bezlotoxumab has no treatment effect on an active CDI episode and should only be administered concurrently with either vancomycin or fidaxomicin for prevention of recurrent CDI [21–23]. Additionally, its high cost (~$4,560 per vial) may prohibit its use in some patients, and its use requires careful consideration, especially in individuals with a history of heart failure [23–25]. Moreover, bezlotoxumab has unfortunately been discontinued as of January 31, 2025 without a given reason, leaving one less treatment option [26]. FMT, the procedure involving reintroducing beneficial gut organisms to restore gut microbial balance [27,28], has been the most effective treatment of CDI treating over 90% of cases with little or no side effects [29–31]. However, FMT has several limitations, including the high cost, risk of infection transmission particularly in the immunocompromised patients, variability in donor material, and unclear long-term safety, and its lack of necessary infrastructure and resources [32–35]. Hence, the need to develop new therapeutics for CDI treatment cannot be overemphasized.

One of the traditional sources for discovering new antibiotic scaffolds is through natural products. Current standard treatments, vancomycin and fidaxomicin, demonstrate minimal systemic absorption and are able to reach the lower gastrointestinal tract (GIT) in high concentrations where *C. difficile* prefers to colonize [36,37]. Many natural products are poorly absorbed due to their size or poor solubility [38,39], which may be ideal for CDI treatment. In a previous drug library screening, the natural product α-mangostin was found to have potent anti-*C. difficile* activity, which is comparable to that of vancomycin [40]. α-mangostin is a plant-sourced xanthone found in the pericarp of the edible fruit mangosteen and has displayed several other biological effects including antimicrobial, antitumor, and antioxidant activities [41–45]. Although α-mangostin has a small molecular weight (410.46 Da), which is could be considered generally more favorable for absorption according to Lipinski’s rule of five, it actually has poor absorption from the GIT [46,47], which is attractive for CDI treatment to accumulate at a sufficient concentration at the infection site. Building upon our previous study, herein, we report the potential of α-mangostin as an anti-CDI therapeutic. Its *in vitro* activity was evaluated against a panel of pathogenic isolates of *C. difficile*. We also assessed its killing kinetics and potency against high *C. difficile* inoculum. Additionally, we investigated α-mangostin’s impact on the bacterial cell membrane integrity. Finally, the anti-*C. difficile* activity of other natural compounds from mangosteen fruits were evaluated in comparison to α-mangostin.

## Materials and methods

### Bacterial strains, reagents, and media

Bacterial strains (S1 Table in S2 File) were obtained from the CDC (Atlanta, GA), the Biodefense and Emerging Infections Research Resources Repository (BEI Resources) (Manassas, VA) and the American Type Culture Collection (ATCC) (Manassas, VA). Media and reagents were purchased commercially: Phosphate-buffered saline (PBS) (Corning, NY), Brain heart infusion broth (BHI) and anaerobic GasPak Sachets (Becton, Dickinson and Company, Sparks, MD), yeast extract (Fisher Scientific, Suwanee, GA), L-cysteine (ThermoFisher Scientific, Waltham, MA), vitamin K1, resazurin, and hemin (Sigma-Aldrich, St. Louis, MO). Drugs were purchased from commercial vendors: α-mangostin (Ambeed: Arlington Heights, IL), vancomycin (Gold Biotechnology, St. Louis, MO), nisin (Cayman Chemical, Ann Arbor, MI), and fidaxomicin (Biosynth Carbosynth, San Diego, CA). Xanthone derivatives derived from *Garcinia mangostana*: garcinone C and β-mangostin (Targetmol, Boston, MA), garcinone D (Ambeed, Arlington Heights, IL), gartanin, 8-desoxygartanin and γ-mangostin (A2B Chem, San Diego, CA), and 3-isomangostin (GlpBio, Montclair, CA) were all purchased commercially.

### Antibacterial activity of α-mangostin against a panel of *C. difficile* clinical isolates

The broth microdilution technique was used to determine the minimum inhibitory concentrations of α-mangostin and control drugs [48–51]. A bacterial solution equivalent to 0.5 McFarland standard was diluted in brain heart infusion supplemented (BHIS) broth to obtain a final bacterial concentration of about 5 × 10^5^ CFU/mL. α-mangostin alongside control antibiotics vancomycin and fidaxomicin, were serially diluted in 96-well plates, and bacterial solution was added. DMSO (1%) was included as a growth control. These plates were then incubated anaerobically at 37 °C for 48 hours. The MIC was determined as the lowest concentration of tested agents that inhibited bacterial growth as observed visually. The concentrations that inhibited 50% and 90% of the strains tested (MIC_50_ and MIC_90_, respectively), were determined. MICs were performed at least in two independent experiments, each containing biological triplicate.

### Time-kill kinetics assay

In order to determine the killing kinetics of α-mangostin, a time kill kinetics assay was performed as previously described [52–54]. *C. difficile* 630 and ATCC BAA-1870 were grown overnight and diluted in sterile BHIS, resulting in a concentration of ~ 10^5^ CFU/mL. Bacteria were subsequently treated with α-mangostin, vancomycin, and fidaxomicin (at 5 × MIC) and then incubated anaerobically at 37 °C. DMSO (1%) was included as a growth control. Aliquots were collected at 0.5, 1, 1,1.5, 2, 6, 12, and 24 hours, serially diluted and subsequently plated onto BHIS agar plates. Spotted agar plates were then placed in anaerobic conditions at 37°C before determining the bacterial CFU. Experiments were performed in triplicate in 2 independent experiments. The data are presented as average of the experiments and the error bars indicate the standard deviation (SD) calculated from the average.

### *C. difficile* inoculum effect on α-mangostin’s activity

The efficacy of α-mangostin against high inoculum sizes of *C. difficile* 630 and 43255 was evaluated, utilizing the broth microdilution assay as previously reported [55–58]. Briefly, standard inoculum (~5 × 10^5^ CFU/mL) and high inoculum sizes (~5 × 10^7^ and 5 × 10^8^ CFU/mL) of *C. difficile* strains were prepared in BHIS broth and tested against α-mangostin and control antibiotics as previously mentioned. DMSO (1%) was included as a growth control. These plates were incubated as previously mentioned and MICs were determined. Experiments were performed in triplicate in at least 2 independent experiments.

### Mechanistic studies and cell membrane permeability assays

#### ATP leakage assay.

To investigate α-mangostin’s impact on the bacterial membrane integrity, the quantity of ATP leaking from *C. difficile* cells treated with α-mangostin was measured as previously reported [59,60]. A logarithmic phase culture of *C. difficile* ATCC 43255 was treated with α-mangostin, nisin, DMSO, or vancomycin (at 5 × MIC), and were incubated anaerobically at 37 °C for 2 hours in triplicate. Vancomycin and DMSO act as negative controls in this assay. Vancomycin demonstrates cell-wall synthesis inhibition [61] rather than membrane activity whilst untreated groups are incubated with the vehicle (DMSO (0.5%)). After incubation, cultures were centrifuged at 10,000 × g for 10 minutes. Supernatants containing leaked ATP were collected and cell pellets were resuspended in pre-reduced BHIS. The amount of ATP in the supernatants (extracellular) and resuspended pellets (intracellular) was measured via BacTiter-Glo Microbial Cell Viability kit (Promega, Madison, Winsconsin), following the manufacturer’s instructions. Luminescence was then determined via Tecan Spark multimode microplate reader. Experiments were performed in triplicate in 2 independent experiments. The data are presented as average of the experiments and the error bars indicate the standard deviation (SD) calculated from the average.

#### DNA leakage assay.

To evaluate whether α-mangostin disrupts the *C. difficile*’s bacterial membrane integrity, the DNA levels were quantified using the nanodrop, after brief incubation with α-mangostin, as previously reported [60,62]. A log-phase culture of *C. difficile* ATCC 43255 was treated with α-mangostin, vancomycin, nisin (at 5 × MIC), or DMSO (at a volume equivalent to that of α-mangostin), and incubated anaerobically for 2 hours. Tubes were centrifuged at 6,000 RPM for 10 minutes. Supernatants were then collected, and the extracellular DNA concentration was measured using NanoDrop One (Thermo Scientific). Experiments were performed in triplicate in 2 independent experiments. The data are presented as average of the experiments and the error bars indicate the standard deviation (SD) calculated from the average.

## Results

### Antibacterial activity of α-mangostin against a panel of *C. difficile* clinical isolates

The anti-*C. difficile* activity of α-mangostin was evaluated against a panel of 30 clinical isolates of *C. difficile* (**Table 1**). α-mangostin inhibited the tested strains at an MIC range of 0.5–2 µg/mL. It inhibited 50% and 90% of the tested strains of *C. difficile* (MIC_50_ and MIC_90_, respectively) at the concentrations of 1 and 2 µg/mL. Remarkably, α-mangostin displayed a similar range of activity to that of the drug of choice vancomycin, which showed an MIC range of 0.5–1 µg/mL. Fidaxomicin demonstrated an MIC range of 0.008–0.06 µg/mL, with MIC_50_ and MIC_90_ values of 0.03 and 0.06 µg/mL.

**Table 1:** MICs (µg/mL) of α-mangostin against 30 clinical strains of *C. difficile.*

*C. difficile* Strains	MICs (µg/mL)		
	α-Mangostin	Vancomycin	Fidaxomicin
*C. difficile* 630	0.5	2	0.03
ATCC 43255	0.5	1	0.06
ATCC 43598	1	1	0.06
ATCC-BAA 1870	1	0.5	0.06
ATCC 1871	2	1	0.03
ATCC 9689	1	0.5	0.016
ATCC 70057	1	1	0.03
NR-49288	2	1	0.06
NR-49302	0.5	0.5	0.016
NR-49304	2	1	0.03
NR-49308	0.5	0.5	0.016
NR-49306	1	0.5	0.03
NR-49310	2	1	0.03
NR-49313	1	1	0.03
NR-49318	1	1	0.03
NR-49319	1	0.5	0.016
AR 1076	1	2	0.06
AR 1078	1	1	0.03
AR 1081	2	0.5	0.03
AR 1082	0.5	1	0.008
AR 1086	0.5	1	0.008
AR 1087	2	0.5	0.03
AR 1093	1	0.5	0.03
AR 1094	1	0.5	0.03
HM-88	2	1	0.06
HM-89	1	1	0.03
HM-745	1	0.5	0.016
NR-32888	2	1	0.03
NR-32884	2	1	0.03
NR-32897	2	1	0.06
**MIC**_**50**_	**1**	**1**	**0.03**
**MIC**_**90**_	**2**	**2**	**0.06**

**MIC**_**50**_: Concentration of test agent which inhibited 50% of tested isolates.

**MIC**_**90**_: Concentration of test agent which inhibited 90% of tested isolates.

### Killing kinetics of α-mangostin against *C. difficile*

**Fig 1:**
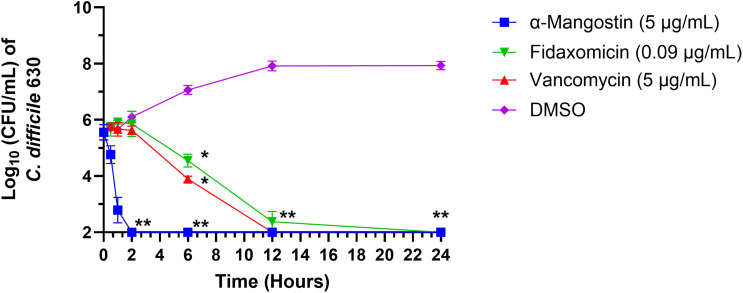
Time-kill kinetics assay of α-mangostin against *C. difficile.* Bacteria were treated with either α-mangostin, vancomycin, fidaxomicin (at 5 × MIC) or DMSO (negative control). Aliquots were taken at the corresponding time points, diluted and plated. The data are presented as log_10_ CFU/mL of bacterial counts at the corresponding time points. The error bars represent standard deviation values for each time point. The data were analyzed via a two-way ANOVA with post-hoc Dunnett’s test for multiple comparisons. Asterisks (****) indicate a statistically significant difference (P < 0.0001) between treatment with test agents as compared to the negative control.

α-mangostin has been previously reported to show a rapid bactericidal activity against MRSA [63]. To assess whether the killing kinetics of α-mangostin are similar against *C. difficile*, a time-kill assay was performed against *C. difficile* 630. α-mangostin (at 5 × MIC) demonstrated a rapid bactericidal activity reducing the bacterial count by about 3 log_10_ CFU/mL and completely eliminating the *C. difficile* count below the limit of detection after 2 hours (**Fig 1**). This rapid killing activity was also demonstrated in the hypervirulent *C. difficile* isolate ATCC BAA-1870 (**S1 Fig in** **S1 File**). Vancomycin reduced the bacterial count by 2 log_10_ CFU/mL within 6 hours and completely cleared the bacterial count after 12 hours. Fidaxomicin (at 5 × MIC) reduced bacterial count by approximately 3 log_10_ CFU/mL within 12 hours with clearance of the bacterial burden 24 hours.

### The impact of *C. difficile* inoculum on the antibacterial activity of α-mangostin

The dependence of the antibacterial activity on the inoculum effect is an important consideration for anti-*C. difficile* therapeutics. Thus, we evaluated the impact of the high *C. difficile* inoculum (~5 × 10^7^ and 5 × 10^8^ CFU/mL), compared with the standard inoculum (~ 5 × 10^5^ CFU/mL), on α-mangostin’s antibacterial activity. The antibacterial activity of α-mangostin against the high inoculum sizes of *C. difficile* ATCC 43255 and *C. difficile* 630 (10^7^ and 10^8^ CFU/mL) was identical to or one-fold higher than its corresponding MICs against the standard inoculum (10^5^ CFU/mL) (**Table 2**), suggesting that its activity was not impacted by increasing the inoculum size. Similarly, the MICs of standard-of-care antibiotics, vancomycin and fidaxomicin were not significantly affected by increasing the *C. difficile* inoculum size (MICs of high inoculums were equal to or one-fold higher than standard inoculum MICs).

**Table 2:** MICs (µg/mL) of α-mangostin and control antibiotics against *C. difficile* clinical isolates at standard (10^5^ CFU/mL) and high (10^7^ and 10^8^ CFU/mL) inoculum sizes.

Test agents	*C. difficile* ATCC 43255			*C. difficile* 630		
	10^5^ CFU/mL	10^7^ CFU/mL	10^8^ CFU/mL	10^5^ CFU/mL	10^7^ CFU/mL	10^8^ CFU/mL
**α-Mangostin**	0.5	1	1	0.5	1	1
**Vancomycin**	1	1	1	1	2	2
**Fidaxomicin**	0.06	0.06	0.06	0.03	0.06	0.06

### Evaluation of the disruptive effect of α-mangostin on the bacterial cell membrane integrity in *C. difficile* cells

**Fig 2:**
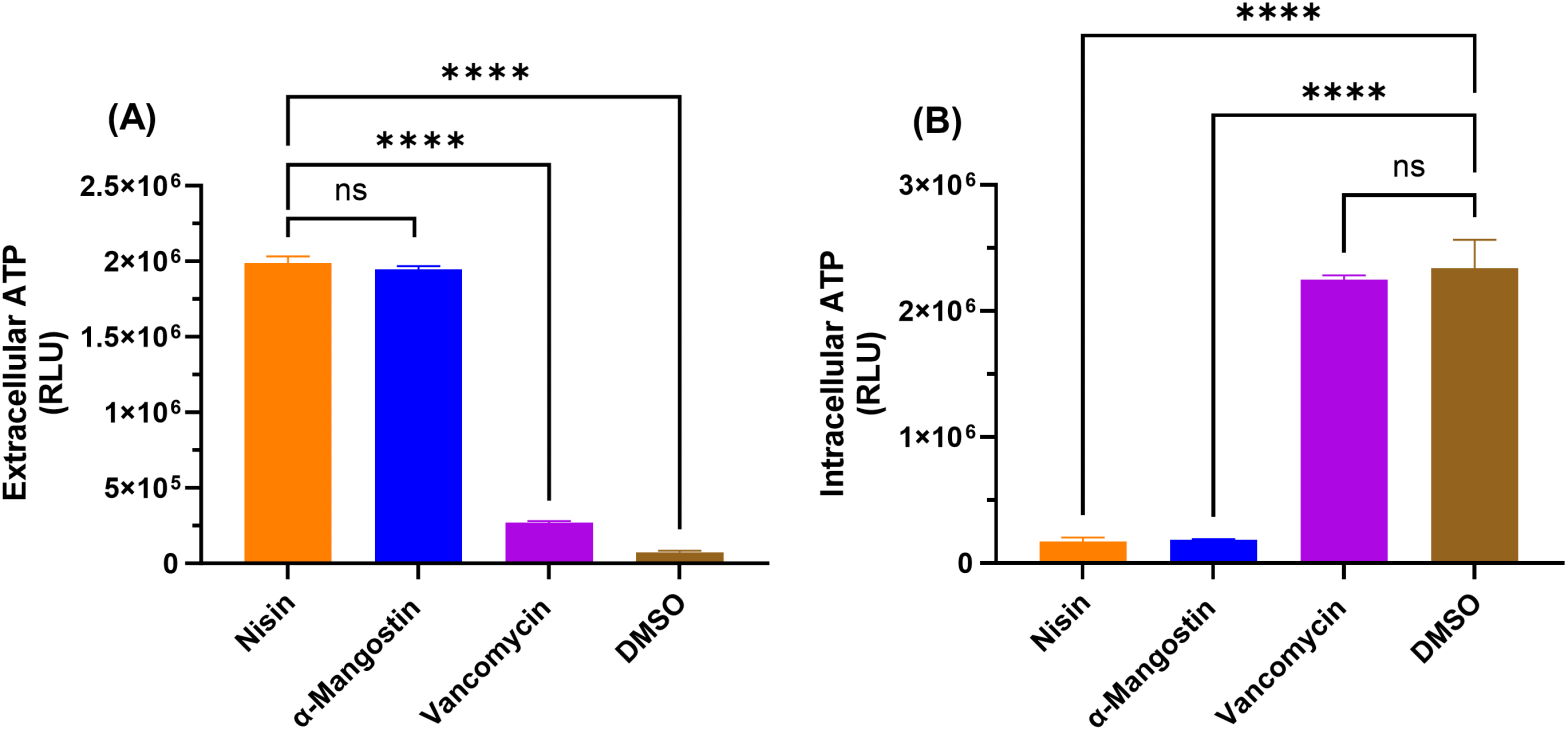
The impact of α-mangostin on the ATP Leakage from *C. difficile* cell membrane. Bacterial cells were exposed to either α-mangostin, nisin (positive control), or vancomycin (negative control) (at 5 × MIC). Relative luminescence units (RLU) representing the amount of ATP measured extracellularly (A) or intracellularly (B). The data were analyzed via a one-way ANOVA test followed by Dunnett’s test for multiple comparisons. Asterisks (*) indicate a statistically significant difference between treatment with test agents as compared to DMSO (untreated); **** (P < 0.0001).

Drugs exhibiting a very rapid bactericidal activity are often associated with disrupting the bacterial cytoplasmic membrane integrity. To determine whether the anti-*C. difficile* activity of α-mangostin is mediated by disrupting the *C. difficile* cytoplasmic membrane, we measured the ATP and DNA leakage after exposure to α-mangostin. Nisin, which is known for its potent membrane-disrupting activity, was included as a positive control, while vancomycin that inhibits bacterial cell wall synthesis without directly targeting the cytoplasmic membrane, was utilized as a negative control. As depicted in **Fig 2A and 2B**, exposure to α-mangostin (at 5 × MIC) for 2 hours resulted in a significant percentage of ATP leakage, which is indicative of increased membrane permeability. The extracellular ATP was found in abundance, as indicated by the relative luminescence units (RLU) (approximately 2 × 10^6^ RLU) with only relatively small amounts of intracellular ATP were detected. This effect was similar to that of the positive control nisin (approximately 2 × 10^6^ RLU for the extracellular ATP, and <5 × 10^5^ RLU for the intracellular ATP). On the other hand, vancomycin, as expected, did not show ATP leakage as indicated by its low RLU for extracellular ATP (<5 × 10^5^ RLU) and high RLU for intracellular ATP (approximately 2 × 10^6^ RLU), which was similar to the untreated control.

**Fig 3:**
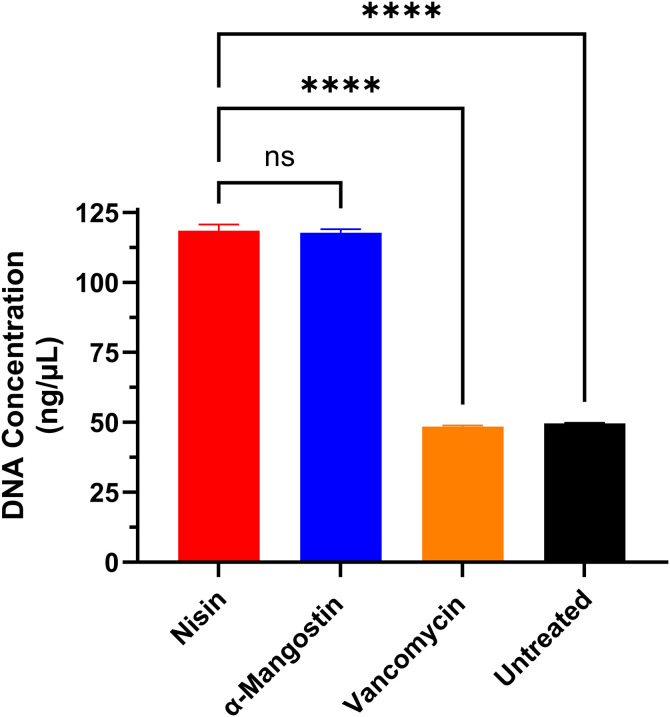
DNA leakage activity of α-mangostin against *C. difficile.* Bacterial cells were exposed to either α-mangostin, nisin (positive control), or vancomycin (negative control) (at 5 × MIC) for 2 hours. The data are presented as released DNA concentration (ng/µL) after exposure to test agents. The data were analyzed via a one-way ANOVA test followed by Dunnett’s test for multiple comparisons. Asterisks (*) indicate a statistically significant difference between treatment with test agents as compared to DMSO (untreated); **** (P < 0.0001).

Further, the DNA levels following the exposure of *C. difficile* ATCC 43255 to α-mangostin (5 × MIC) provided additional evidence of its membrane disruptive activity. Nisin, the positive control, caused intracellular DNA leakage (~ 100 ng/µL). α-mangostin displayed a similar effect to nisin inducing DNA leakage resulting in DNA concentration of ~ 100 ng/µL (**Fig 3**). Vancomycin, as expected, did not lead to significant DNA leakage (~20 ng/µL), which was similar to the negative control, DMSO. Altogether, these results confirm that the anti-*C. difficile* activity of α-mangostin is associated with disruption of the cell membrane integrity.

### Activity of related xanthones from mangosteen fruits against *C. difficile*

As a final step, we tested other xanthone products from mangosteen fruits and whether similarly structured compounds maintained their efficacy against *C. difficile*. We found that β-mangostin, γ-mangostin and 8-deoxygartanin displayed comparable activity to that of α-mangostin, with MICs of 1 µg/mL. 3-Isomangostin showed slightly higher MIC than α-mangostin (MIC = 2 µg/mL). Garcinone C and garcinone D were 8-fold less active than α-mangostin (MIC = 4 µg/mL), while gartanin was the least potent among the tested derivatives with a MIC of 8 µg/mL (**Table 3**). MIC assays were carried out in duplicate in 2 independent experiments to confirm results.

**Table 3:** The anti-*C. difficile* activity of other natural compounds from mangosteen fruits and structurally related xanthones purchased commercially.

Compound	*C. difficile* 630 MICs (µg/mL)
Garcinone C	4
Garcinone D	4
Gartanin	8
8-deoxygartanin	1
3-Isomangostin	2
β-Mangostin	1
γ-Mangostin	1
α-Mangostin	0.5
Vancomycin	1
Fidaxomicin	0.03

## Discussion

Given the substantial health burden of CDI, and the limitations of the currently available therapeutics, new effective therapies are critically needed. An ideal anti-*C. difficile* antibiotic should have potent activity against *C. difficile* and be poorly absorbed from the intestine to accumulate at the site of infection in the colon and large intestine at concentrations capable of clearing infection. Consequently, we previously screened a library of natural products against *C. difficile* [40]. We selected these natural products because they are commercially available and some of them are generally poorly absorbed by the GIT [38,39], and have not been widely screened against *C. difficile*. From this screening, α-mangostin was found to have potent anti-*C. difficile* activity comparable to that of vancomycin whilst killing far more rapidly with little to no cytotoxicity as demonstrated in our cytotoxicity assay against colonic epithelial cells (Caco-2) (S2 Fig in S1 File).

α-mangostin is a natural product xanthone isolated from mangosteen fruits’ pericarp that has a wide-range of biological activities including antimicrobial activity against Gram-positive bacterial and fungal pathogens such as *Candida albicans* [64]. α-mangostin is also non-toxic to cells [44,65]. Whilst α-mangostin is known for all of these beneficial effects, its extremely poor pharmacokinetics severely restrict its widespread application to a topical treatment or an antiseptic solution [46,66]. However, the poor absorption of α-mangostin is advantageous for *C. difficile* to accumulate at high concentrations at the site of infection.

In this study, α-mangostin’s activity was evaluated against a panel of pathogenic *C. difficile* strains. The natural product demonstrated very potent activity similar to vancomycin, with an MIC_50_ of 0.5 μg/mL. Interestingly, the drug also maintained the same potency against the different ribotypes of *C. difficile*, including the hypervirulent ribotypes such as 027 (ATCC-BAA 1870, AR-1076, etc.) and 078 (ATCC 43255, NR-49310, etc.). *C. difficile* ribotype 027 is recognized as the most significant hypervirulent strains, linked to numerous global outbreaks and high mortality rates [67]. Additionally, ribotype 087 strains are associated with a rise in community-acquired CDI cases and have also shown connections to increased disease severity and mortality [68].

In order to understand how quickly α-mangostin could kill *C. difficile*, a time-kill kinetics assay was performed. α-mangostin exerted rapid bactericidal activity, completely eliminating the high *C. difficile* count within 2 hours consistent with a previous study done on MRSA, another Gram-positive pathogen [63]. Rapid bactericidal activity is desirable for anti-CDI therapeutics as it could lessen the risk of complications like severe colitis and reduce the emergence of bacterial resistance by facilitating rapid clearance of *C. difficile* [69–72]. Vancomycin showed a gradual decline in the *C. difficile* count, consistent with previous reports [73–75]. Fidaxomicin demonstrated a bactericidal activity against the tested strain, in coincidence with previous studies [40,76].

An important hallmark of *C. difficile* is its ability to form spores. Spores are metabolically inactive yet highly resistant to conventional disinfectants, allowing them to survive for extended periods and persist in the environment. After ingestion by susceptible individuals, these spores germinate in response to bile acids in the small intestine, forming vegetative cells that produce toxins and cause disease. Moreover, spores that remain in the intestine can survive treatment and later germinate, contributing to recurrent infection [77,78]. Hence, we have conducted a spore inhibition assay for α-mangostin and it did not show any significant inhibition of *C. difficile* spore formation (S3 Fig in S1 File).

*C. difficile* commonly establishes high-level colonization in the intestinal tract. Studies have shown that bacterial loads in the cecal and fecal material of infected mice typically range between 10⁶ and 10^7^ CFU per gram [79] and may reach similar or higher levels in human patients [80]. This highlights the relevance of the inoculum effect when assessing the efficacy of anti-*C. difficile* agents, particularly for agents with limited systemic absorption like α-mangostin, where it accumulates in the GIT and acts directly on *C. difficile* populations. Despite this, in the standard antimicrobial susceptibility assays, a lower bacterial inoculum (~10⁵ CFU/mL) is used. To address this discrepancy, we assessed the influence of a higher *C. difficile* inoculum sizes (~5 × 10^7^ and 5 × 10^8^ CFU/mL) compared to the standard inoculum (5 × 10⁵ CFU/mL) on the antibacterial performance of α-mangostin. The natural product’s anti-*C. difficile* activity was not impacted by increasing the inoculum size. Similarly, vancomycin and fidaxomicin MICs, in agreement with previous studies [55,81], were not affected by increasing the inoculum size.

This rapid bactericidal activity of α-mangostin is indicative of having bacterial membrane disruptive activity resulting in rapid lysing of bacterial cells. Exposure of bacterial cells to membrane disruptive agents was reported to cause DNA leakage and ATP depletion, which arises from uncoupling of ATP biosynthesis or from membrane permeabilization and cell leakage [82]. Therefore, to evaluate the membrane disruptive activity, an ATP and DNA leakage assay was performed. Nisin, an antibiotic peptide produced by *Lactococcus lactis*, was utilized as a control in these experiments for its ability to rupture bacterial cytoplasmic membrane [83–85]. When compared, nisin and α-mangostin both have high levels of ATP leakage, whilst having very little ATP intracellularly. They also generated a significant DNA leakage. Vancomycin and untreated groups demonstrated the opposite effect where low amounts of ATP and DNA were found extracellularly and high amounts were found intracellularly.

Finally, the anti-*C. difficile* activity of other xanthone extracts from mangosteen fruits was evaluated in comparison to α-mangostin. Xanthones have previously demonstrated very poor absorption and have fairly similar structures to α-mangostin prompting further investigation of other family members from the same sources that may be more potent or better fit as an anti-*C. difficile* compound [86,87]. Among the tested compounds, α-mangostin exhibited the strongest activity with an MIC value of 0.5 µg/mL, suggesting that the presence of hydroxyl groups at positions C-1, C-3, and C-6, and a methoxy group at C-7 is crucial for its high activity. β-mangostin, γ-mangostin, and 8-deoxygartanin, which have an MIC of 1 µg/mL, demonstrated that subtle changes, such as the position of methoxy groups (as in β-Mangostin), the presence of an extra hydroxyl group (as in γ-mangostin), or the absence of a hydroxyl group (as in 8-deoxygartanin), slightly reduced their activity. 3-Isomangostin had an MIC of 2 µg/mL, suggesting that the rearrangement of functional groups may lead to moderate reduction of activity (S2 Table in S2 File). As the structures significantly deviated from α-mangostin (increased substitution with bulky groups or loss of key hydroxyl functionalities), the activity was reduced, as seen in garcinone C and D (MIC of 4 µg/mL) and gartanin (MIC of 8 µg/mL). γ and β-mangostin have demonstrated activity against other microorganisms such as *Leptospira* [88,89] and anticancer effects [90,91]. Garcinone C, D were reported to have anti-leptospiral action [88].

In conclusion, this study highlights α-mangostin as a potent inhibitor for *C. difficile*. It exhibited rapid bactericidal activity, and its antibacterial activity was not affected by the high *C. difficile* inoculum sizes. The rapid bactericidal activity was shown to be mediated by disruption of the bacterial cytoplasmic membranes leading to ATP and DNA leakage. This suggests that α-mangostin could significantly disrupt the intestinal microbiota, which could be considered a limitation in the context of CDI therapy, where preservation of the gut microbiota is critical. Nevertheless, the anti-commensal activity of α-mangostin will be comprehensively evaluated in an *in vivo* microbiome analysis in future studies. Another limitation of the study is the lack of the efficacy of α-mangostin in a CDI mouse model, which will be conducted in our future studies to validate the *in vitro* findings in *in vivo* model. Altogether, these findings indicate that α-mangostin warrants further investigation including validation of the *in vitro* findings in *in vivo* mouse models of CDI.

## Supporting information

S1 File Supporting and supplementary figures. Additionally includes methodology and accompanying captions.
(DOCX)

S2 File Supporting and supplementary tables. Tables of strains utilized and related xanthone compounds’ chemical structures and molecular weights.
(DOCX)

S3 File Raw data for figures in excel format. (XLSX)
