# Supplementary material for: Evaluation of the antibacterial activity of the natural product α-mangostin against Clostridioides difficile
Source: PLoS One. 2026 Feb 5;21(2):e0341857. doi: 10.1371/journal.pone.0341857 (PMC12875497; doi:10.1371/journal.pone.0341857)
Supplement: S1 File — Additionally includes methodology and accompanying captions. (DOCX) [file pone.0341857.s001.docx]

**Methods**

**MTS/PMS Cytotoxicity assay**

The potential cytotoxicity of the compounds were evaluated against caco-2 cells as previously described [1]. Caco-2 cells were cultured in Eagle’s minimal essential medium (MEM) supplemented with 20% FBS and 1% penicillin/streptomycin were grown to sufficient confluency at 37°C in the presence of 5% CO_2_. Cells were exposed to trypsin to detach cells from tissue culture flasks and seeded onto treated 96-well plates till 100% confluent. Compounds, including vancomycin, fidaxomicin and α-mangostin, were incubated for 24 hours in the previously mentioned conditions. Old media was removed and treated cells were further incubated for 4 hours with MEM with 20% trazolium compound [3-(4,5-dimethylthiazol-2-yl)-5-(3-carboxymethoxyphenyl)-2-(4-sulfophenyl)-2H-tetrazolium] (MTS reagent) and 1% phenazine methosulfate (PMS). Color change may be measured via absorbance on a plate reader at 490 nm.

**Sporulation Inhibition Assay**

Spore formation, or sporulation, was assessed as previously described [2] with minor modifications. Log-phase cultures of *C. difficile* ATCC BAA-1870 were incubated at sub-inhibitory concentrations (0.5x MIC) for 6 days anaerobically at 37°C. Aliquots were obtained, serially diluted, and plated onto BHIS agar supplemented with 0.1% taurocholic acid, a *C. difficile* germinant, to evaluate vegetative and spore counts combined. The remaining solution was resuspended in PBS after brief centrifugation and heat shocked at 70°C for 40 minutes to kill remaining vegetative cells. Pellets were then stored overnight at 4°C and serially diluted and spotted onto BHIS agar with 0.1% taurocholic acid to asses remaining spores via colony counting.





**Supplementary Figure 1. Time-kill kinetics assay of** **α-mangostin against hypervirulent clinical isolate of *C. difficile* ATCC BAA-1870.** Bacteria were treated with either α-mangostin, vancomycin, fidaxomicin (at 5× MIC) or DMSO (negative control). Aliquots were taken at the corresponding time points, diluted and plated. The data are presented as log_10_ CFU/mL of bacterial counts at the corresponding time points. The error bars represent standard deviation values for each time point.





**Supplementary figure 2. MTS cytotoxicity assay of α-mangostin versus caco-2 cells**

Cells were incubated for 24 hours in the presence of α-mangostin and 6.4 µL of vehicle DMSO, equivalent to the volume of α-mangostin used at the starting concentration of 32 µg/mL. Color change was observed via absorbance at 490 nm on a microplate reader to identify cytotoxicity at a given concentration.





**Supplementary figure 3. Sporulation inhibition activity of α-mangostin against *C. difficile* ATCC-BAA 1870.** Control antibiotics vancomycin and fidaxomicin were utilized as negative and positive controls respectively. Bacteria were incubated with compounds at half their MIC for 6 days and serially diluted onto plates to evaluate bacterial and spore counts. Error bars represent standard deviation values from triplicates. Asterisks (*) denote significant differences between total and spore count for each compound via two-way ANOVA with *post hoc* Dunnett’s test for multiple comparisons (P< 0.05)

**References**

1. Pal R, Seleem MN. Discovery of a novel natural product inhibitor of Clostridioides difficile with potent activity in vitro and in vivo. PLOS ONE. 2022;17(8):e0267859. doi: 10.1371/journal.pone.0267859.

2. Abutaleb NS, Seleem MN. Repurposing the Antiamoebic Drug Diiodohydroxyquinoline for Treatment of Clostridioides difficile Infections. Antimicrobial Agents and Chemotherapy. 2020;64(6):10.1128/aac.02115-19. doi: doi:10.1128/aac.02115-19.
