# Supplementary material for: Evaluation of the antibacterial activity of the natural product α-mangostin against Clostridioides difficile
Source: PLoS One. 2026 Feb 5;21(2):e0341857. doi: 10.1371/journal.pone.0341857 (PMC12875497; doi:10.1371/journal.pone.0341857)
Supplement: S2 File — Tables of strains utilized and related xanthone compounds’ chemical structures and molecular weights. (DOCX) [file pone.0341857.s002.docx]

**Supplementary Table 1. Full description, sources, and characteristics of *C. difficile* strains.**

| No. | *C. difficile Strain ID* | Alternate Designation | Source | Characteristics |
| --- | --- | --- | --- | --- |
| 1 | **ATCC 630** | *Clostridium difficile* BAA-1382 | Sourced from ATCC. | Positive for *tcdA, tcdB*. Negative for *cdtB*. |
| 2 | **ATCC 43255** | *Clostridium difficile* Isolate VPI 10463 | Sourced from ATCC. Isolated from abdominal wound | Positive for *tcdA, tcdB*. Negative for *cdtB*. |
| 3 | **ATCC 43598** | *Clostridium difficile* Isolate 1470 | Sourced from ATCC. Isolated from Belgium from stool of asymptomatic neonate. | Positive for *tcdA, tcdB*. Negative for *cdtB*. |
| 4 | **ATCC-BAA 1870** | *Clostridium difficile* Isolate 4118 | Sourced from ATCC. Clinical Isolate (Ribotype – 027) | Positive for *tcdA, tcdB,* and *cdtB* |
| 5 | **BAA 1871** | *Clostridium difficile* strain 4111 | Sourced from ATCC. | Positive for *tcdA, tcdB*. Negative for *cdtB*. |
| 6 | **ATCC 9689** | *Clostridioides difficile* strain 90556-M6S | Sourced from ATCC. Reference strain. | Positive for *tcdA, tcdB*. Negative for *cdtB*. |
| 7 | **ATCC 70057** | *Clostridioidies difficile* strain VPI 11186 | Sourced from ATCC. | Negative for *tcdA, tcdB,* and *cdtB*. |
| 8 | **NR-49288** | *Clostridium difficile* Isolate 20110870 | Sourced from BEI Resources.  Isolated from a young adult female with HA CDI. | Positive for *tcdA, tcdB, tcdC* and *cdtB*. |
| 9 | **NR-49302** | *Clostridium difficile* Isolate 201111075 | Sourced from BEI Resources.  Isolated from an elderly male with HA CDI. | Positive for *tcdA, tcdB, tcdC* and *cdtB*. Positive for CDT |
| 10 | **NR-49304** | *Clostridium difficile* Isolate 20120956 | Sourced from BEI Resources.  Isolated from older male patient with HA CDI. | Positive for *tcdA, tcdB, tcdC.* Negative for CDT. |
| 11 | **NR-49306** | *Clostridium difficile* Isolate 20110997 | Sourced from BEI Resources.  Isolated from an elderly male patient with CA CDI. | Positive for *tcdA, tcdB, tcdC.* Negative for CDT. |
| 12 | **NR-49308** | *Clostridium difficile* Isolate 20120166 | Sourced from BEI Resources.  Isolated from an elderly female with CA CDI. | Positive for *tcdA, tcdB, tcdC.* Negative for CDT. |
| 13 | **NR-49310** | *Clostridium difficile* Isolate 20110986 | Sourced from BEI Resources.  Isolated from a young male patient with CA CDI. | Positive for *tcdA, tcdB, tcdC.* Positive for CDT. |
| 14 | **NR-49313** | *Clostridium difficile* Isolate 20110963 | Sourced from BEI Resources.  Isolated from an elderly female with HA CDI. | Positive for *tcdA, tcdB, tcdC.* Negative for CDT. |
| 15 | **NR-49318** | *Clostridium difficile* Isolate 20110973 | Sourced from BEI Resources.  Isolated from a pediatric female patient with CA CDI. | Positive for *tcdA, tcdB, tcdC.* Negative for CDT. |
| 16 | **NR-49319** | *Clostridium difficile* Isolate 20110992 | Sourced from BEI Resources.  Isolated from an elderly male with CA CDI. | Positive for *tcdA, tcdB, tcdC.* Negative for CDT. |
| 17 | **CDI-10** | *Clostridium difficile* Isolate 1076 | Sourced from the CDC. Isolated from unknown patient in 2016, USA. | Positive for *tcdA, tcdB,* Negative for CDT. |
| 18 | **CDI-12** | *Clostridium difficile* Isolate 1078 | Sourced from the CDC. Isolated from unknown patient in 2016, USA. | Positive for *tcdA, tcdB,* Negative for CDT. |
| 19 | **CDI-15** | *Clostridium difficile* Isolate 1081 | Sourced from the CDC. Isolated from unknown patient in 2016, USA. | Positive for *tcdA, tcdB,* Negative for CDT. |
| 20 | **CDI-16** | *Clostridium difficile* Isolate 1082 | Sourced from the CDC. Isolated from unknown patient in 2016, USA. | Positive for *tcdA, tcdB,* Negative for CDT. |
| 21 | **CDI-20** | *Clostridium difficile* Isolate 1086 | Sourced from the CDC. Isolated from unknown patient in 2016, USA. | Positive for *tcdA, tcdB,* Negative for CDT. |
| 22 | **CDI-21** | *Clostridium difficile* Isolate 1087 | Sourced from the CDC. Isolated from unknown patient in 2016, USA. | Positive for *tcdA, tcdB,* Negative for CDT. |
| 23 | **CDI-27** | *Clostridium difficile* Isolate 1093 | Sourced from the CDC. Isolated from unknown patient in 2016, USA. | Positive for *tcdA, tcdB,* Positive for CDT. |
| 24 | **CDI-28** | *Clostridium difficile* Isolate 1094 | Sourced from the CDC. Isolated from unknown patient in 2016, USA. | Positive for *tcdA, tcdB,* Positive for CDT. |
| 25 | **HM-88** | NAP07 (CDC#2007054) | Sourced from BEI. Reference genome for human microbiome project (HMP). | Negative for *tcdA, tcdB,* Negative for CDT. |
| 26 | **HM-89** | NAP08 (CDC#2007019) | Sourced from BEI. Reference genome for HMP. | Negative for *tcdA, tcdB,* Negative for CDT. |
| 27 | **HM-745** | 70-100-2010 | Sourced from BEI Resources. Isolated from a patient with diarrhea. Reference for HMP. | Positive for *tcdA, tcdB,* Negative for CDT. |
| 28 | **NR-32884** | *Clostridium difficile* P3 | Sourced from BEI Resources. Isolated from fecal material of a human patient with CDI. | Positive for *tcdA, tcdB,* |
| 29 | **NR-32888** | *Clostridium difficile* P8 | Sourced from BEI Resources. Isolated from a human patient with CDI. | Positive for *tcdA, tcdB,* |
| 30 | **NR-32897** | *Clostridium difficile* P21 | Soured from BEI Resources. Isolated from fecal material of a human patient with relapsing CDI. | Positive for *tcdA, tcdB,* |

**ATCC**: The American Type Culture Collection

**BEI Resources:** The Biodefense and Emerging Infections Resources Repository

**CDC:** The Center for Disease Control and Prevention

**HA:** Hospital acquired

**CA:** Community acquired

**HMP:** Human Microbiome Project

**Supplementary Table 2. Structures and molecular weights of xanthones related to α-Mangostin evaluated.**

| **Compound** | **Structure** | **Molecular Weight (g/mol)** |
| --- | --- | --- |
| Garcinone C | 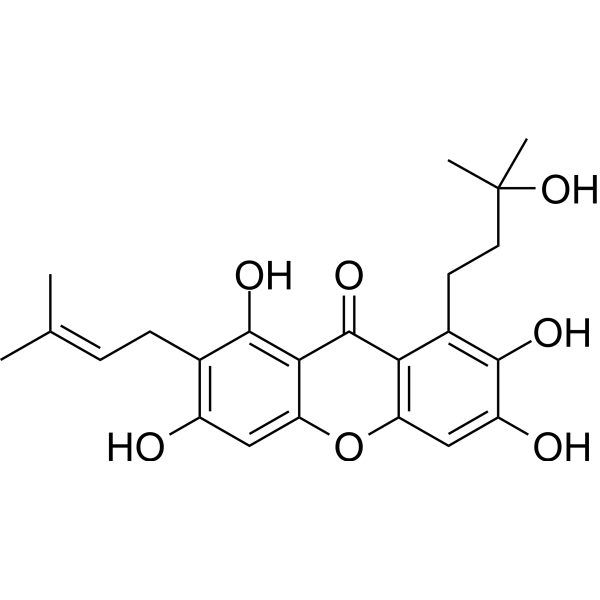 | 414.45 |
| Garcinone D | 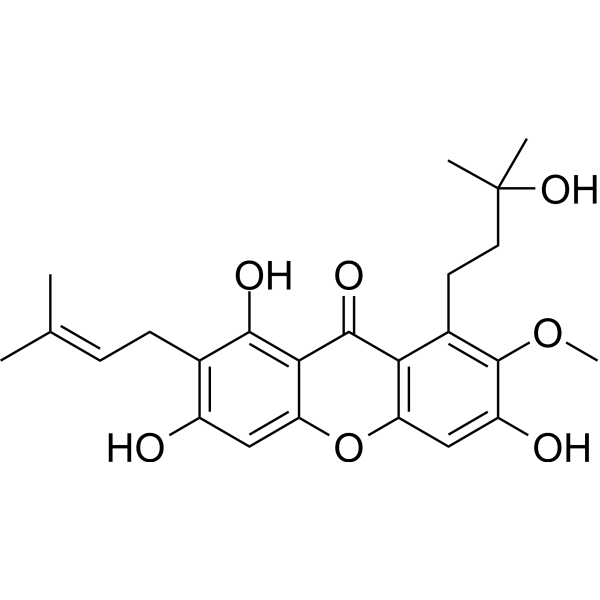 | 428.47 |
| Gartanin | 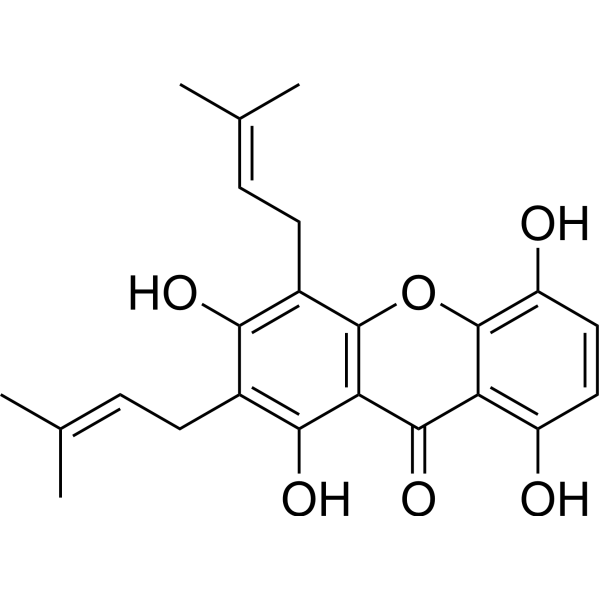 | 396.43 |
| 8-deoxygartanin | 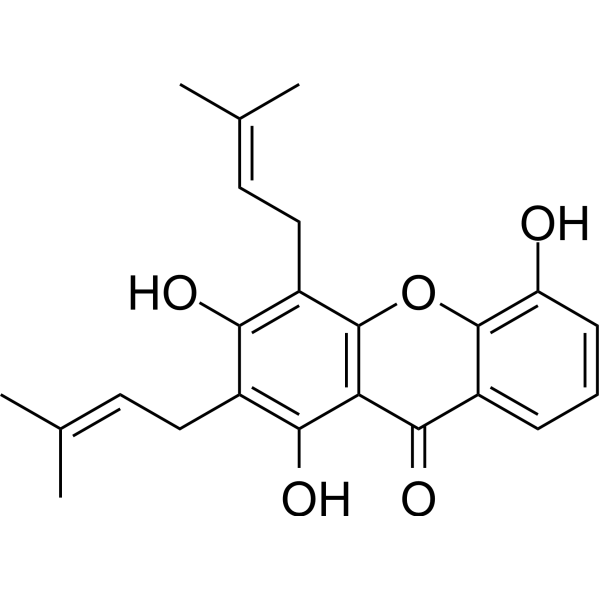 | 380.43 |
| 3-Isomangostin | 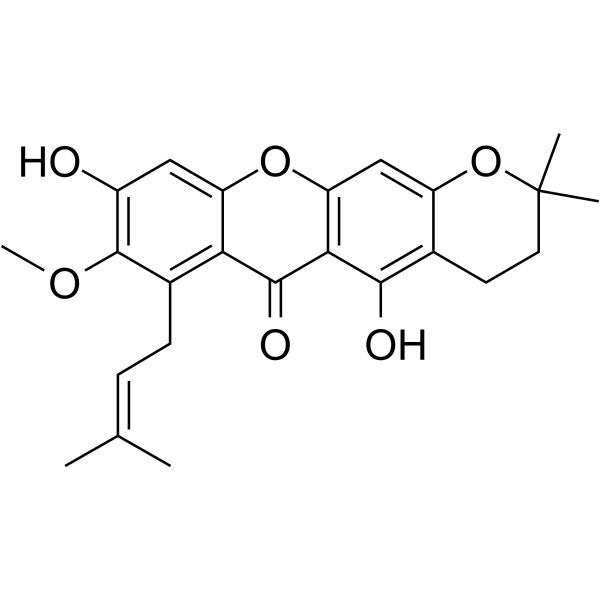 | 410.5 |
| β-Mangostin | 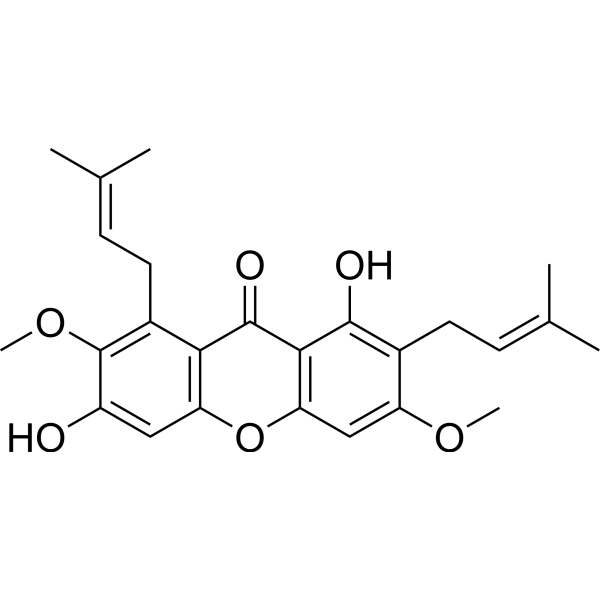 | 424.49 |
| γ-Mangostin | 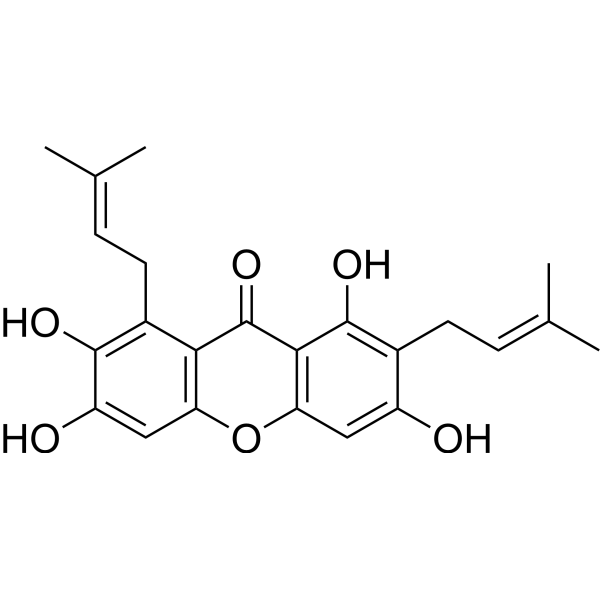 | 396.43 |
| α-Mangostin | 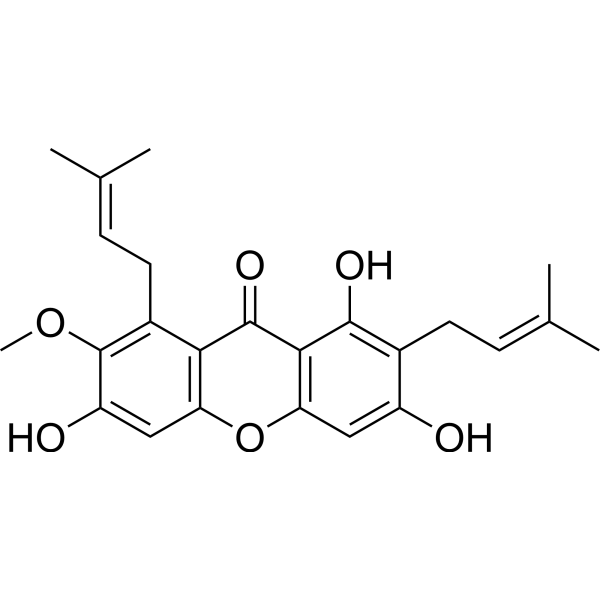 | 410.46 |
| Vancomycin | - | 1449.25 |
| Fidaxomicin | - | 1058.04 |
